# An update on treatment options for primary sclerosing cholangitis 

**Published:** 2020

**Authors:** Shahrokh Iravani, Arash Dooghaie-Moghadam, Niloofar Razavi-Khorasani, Bobak Moazzami, Amirreza Dowlati Beirami, Alireza Mansour-Ghanaei, Keivan Majidzadeh-A, Azim Mehrvar, Alireza Khoshdel, Mohssen Nasiri Toosi, Amir Sadeghi

**Affiliations:** 1 *Research Center for Cancer Screening and Epidemiology, AJA University of Medical Sciences, Tehran, Iran *; 2 *Gastroenterology and Hepatobiliary Research Center, AJA University of Medical Sciences, Tehran, Iran *; 3 *Liver Transplantation Research Center, Tehran University of Medical Sciences, Tehran, Iran*; 4 *Gastroenterology and Liver Diseases Research Center, Research Institute for Gastroenterology and Liver Diseases, Shahid Beheshti University of Medical Sciences, Tehran, Iran*

**Keywords:** Inflammatory bowel disease, Primary sclerosing cholangitis (PSC), Management, Vancomycin, Cholestasis, Cholangitis

## Abstract

Primary sclerosing cholangitis is a chronic cholestatic liver disease defined by strictures of the biliary tree which could ultimately lead to liver cirrhosis and cholangiocarcinoma. Although the exact underlying etiology of this disorder is not fully understood, the pathology is believed to be caused by immune mediated mechanisms. Growing body of evidence suggests several treatment modalities mainly focusing on the inflammation aspect of this disorder. However, there is still no consensus regarding the best treatment option for these patients. Thus, the present study aimed to review the current treatment options for patients with primary sclerosing cholangitis.

## Introduction

 Primary Sclerosing Cholangitis (PSC) is a rare but serious, chronic cholestatic fibroinflammatory liver disease characterized by progressive and multifocal fibrosis of the biliary system, which typically results in cirrhosis and fibrotic liver diseases (1-3). PSC is associated with several comorbidities such as Inﬂammatory bowel disease (IBD), which results in a phenotypically different disease, PSC-IBD, which differs from PSC in management. PSC also significantly increases the risk of developing malignant comorbidities such as cholangiocarcinoma (CCA), hepatocellular carcinoma (HCC), gallbladder carcinoma (GBC), and colorectal carcinoma (CRC) (4). 

Prevalence of PSC differs from 1 to 16 per 100,000 in different societies, and its incidence differs from 1 to 1.3 cases per 100,000 (4, 5). Approximately 70% of patients with PSC have underlying IBD, and prevalence of PSC-IBD has been estimated 24 per 100,000, though the statistics are not accurate because of the varying diagnostic criteria (5). 

The prolonged duration of IBD in PSC-IBD patients seemingly increases the risk of developing CCA and CRC (6, 7). Annually, 0.5% to 1.5% of patients with PSC develop CCA with the lifetime incidence of GBC and HCC in patients with PSC estimated to be 3%-14% and 0.3%-8%, respectively. There is insufficient data about pancreatic cancer, but it is suggested to be 14 times higher than the healthy population in PSC patients (4). Altogether, the frequency of hepatobiliary malignancies in patients with PSC is estimated 13% (8).

Due to PSCs complications and comorbidities as well as their high rate of mortality, early diagnosis of it and its comorbidities is important as it provides more time to determine the best tactics for treatment of PSC, prevention and/or early detection of the comorbidities, as well as management of malignancies in early stages (4, 5, 9, 10). Clinical symptoms such as fatigue, pruritus, and jaundice can be a result of PSC but the major problem with PSC diagnosis is that it is often not clinically symptomatic. Further, unlooked-for findings of impaired biliary ducts on imaging or asymptomatic elevation of hepatic biomarkers such as alkaline phosphatase are more common diagnostics in PSC diagnosis (1). Finally, the diagnosis of PSC can be confirmed by cholangiography. On the other hand, many experts recommend regular magnetic resonance imaging with magnetic resonance cholangiopancreatography (MRI/MRCP) which potentially improves the outcome for CCA, as the most common malignancy of PSC (1, 3, 4, 11). In this article, we will review the potential and current drug therapies for treating and modifying the complications of PSC and their efficacy based on clinical trial studies.

**Pathophysiology of PSC**

The main causes of PSC have remained unknown so far. According to the results of the prevalence of PSC, environmental factors and genetic polymorphisms may play an important role in the disease prevalence. Recently, part of the abnormal composition of gut flora, genetic susceptibility, and immune system dysfunction have attracted researchers' attention (12-14). Cholangitis or inflammation of the bile ducts is the main characteristic of PSC disease. Development of these inflammatory reactions can induce scar formation in these ducts and subsequently, the biliary tract narrows; eventually, the bile ducts get blocked with the PSC progression (15).

Blocked bile ducts lead to an increased susceptibility to progressive biliary fibrosis, biliary cirrhosis, and eventually liver failure. Inflammation of the bile ducts and scars in these ducts can make the patient susceptible to cholangiocarcinoma. Also, with chronic inflammation of the bile ducts, the risk of gallbladder epithelial dysplasia and gallbladder neoplasia increases. Sometimes an unusual itching is reported in patients with PSC due to bile salt retention and endogenous opioid ligand accumulation. In patients with PSC, due to biliary obstruction, the amount of conjugated bile acids in the intestine decreases. These conjugated bile acids are essential for lipid uptake and lipid-soluble compounds. Thus, in patients with PSC and reduced conjugated bile acids, absorption of fat-soluble vitamins (A, D, E, and K) diminishes, whereby the clinical symptoms associated with it can occur (16-18).

**Genetic and Immune Factors in PSC**

As previously discussed, there are several genetic and immune factors associated with PCS occurrence, progression, and response to treatment (19-22). Generally, it seems the most significant part of this influence is related to human leukocyte antigen (HLA) haplotypes (21, 23-26). For example, *HLA-B8*, *HLA-DR3* (26), *HLA-B*, and *HLA-DRB1* (27) have been shown to be associated with the risk of PSC development. In the liver transplant setting, donors with HLA-DRB1*07 increased the risk of graft failure, while the presence of HLA-DQB1*03, HLA-DRB1*04, and HLA-DQB1*07 in the recipient may have protective roles (28). 

**PSC treatment**

Unfortunately, PSC pathogenesis is poorly understood; thus, PSC is generally considered as an idiopathic disease (2, 29) and there are few therapies available for it. Generally, the most effective treatment of PSC is liver transplantation, but there are also studies suggesting that medication therapy may have a positive effect in modifying the disease (2, 30).

**Liver transplantation**

Liver Transplantation is the only effective treatment proved so far for patients with advanced PSC. The usual time from diagnosis to liver transplantation is within the range of 20 to 25 years. Note that patients with PCS who have undergone liver transplant treatment are prone to acute and chronic cellular rejection (16, 31, 32). On the other hand, there is a significant recurrence of PSC (rPSC) in patients after orthotopic liver transplantation. Reportedly, PCS recurs in 30 to 50% of these individuals. With the recurrence of the disease in some patients, liver transplantation is required again. Studies have also shown that recurrence of PSC can be seen more in patients with PSC-IBD. Nevertheless, liver transplantation is the only treatment for patients with advanced PCS (16, 28, 31, 32). It has also been suggested that patients at the end stage of liver disease (Mayo risk score > 15) should be referred for liver transplantation (33).

Although HLA serotyping is not considered to have a significant influence on the outcome of PSC transplantation, studies suggest that there is an association between HLA serotyping and success of transplantation (19, 28, 34). For example *HLA-B7*, *HLA-B57*, *HLA-B75*, *HLA-DR13*, HLA-DQB1*03, HLA-DRB1*04, and HLA-DQB1*07, in the recipient and HLA-B55, HLA-B58, HLA-DRB1*07, and *HLA-DR8* in the donor are associated with failure of liver transplantation treatment (28, 34).

**Drug therapy of PSC**

Although the pathogenesis of PSC has not been completely understood, there are some hypotheses of possible contributors such as intestinal microbiota (35, 36), immunological pathways (37, 38), and genetics (39-41). On the other hand, although there is no specific treatment for PSC, many studies have reported that some immunosuppressants and immunomodulatory drugs, antibiotics, and anti-inflammatory drugs can help control the disease and its complications (30, 42). Studies are suggesting that controlling and normalizing levels of alkaline phosphatase in the long-term would improve survival and reduce the risk of requiring liver transplantation (43, 44). 

**Anti-pruritus drugs**

About half of patients diagnosed with PSC are asymptomatic at presentation (1, 11). However, in some cases patients complain of pruritus due to extrahepatic cholestasis. Pruritus can be extremely difficult to treat and often present at night. It has also been shown that histamine levels are also significantly elevated in PSC patients (102). Several studies have attempted to examine several drugs for pruritus management such as cholestyramine, Ursodeoxycholic acid, and rifampicin (47, 49). Recently, the use of melatonin has also been gaining attention in the management of gastrointestinal disease as well as anti-pruritus effect. It has been hypothesized that melatonin could reduce the itching among patients with chronic liver disease (103-104). The exact underlying mechanism of action of this agent is not yet fully understood. Although it has been suggested that melatonin could act via immunomodulatory, anti-inflammatory, and antioxidative effects. No large studies have been conducted on the use of this drug as a possible anti-pruritus agent of patients with PSC. Thus, future prospective randomized clinical trial studies are warranted to elucidate its role in reducing itching in these patients.

**Ursodeoxycholic acid**

Ursodeoxycholic acid (UDCA) is a derivative of chenodeoxycholate. It is a hydrophilic mammalian bile acid and the most extensively studied of all medical treatments for PSC (45, 46). Generally, UDCA is used for the treatment of cholestatic liver diseases. It acts mostly through protecting cholangiocytes, stimulating hepatobiliary secretion, and protecting hepatocytes against bile acid-induced apoptosis (47). On the other hand, UDCA is genotoxic, exerts aneugenic activity, and inhibits enzymes and processes such as DNA repair, p53, phagocytosis, and induction of nitric oxide synthetase (48). Although UDCA is the most commonly used and the most commonly studied drug for PSC, four meta-analyses of clinical trials indicated that despite the fact that UDCA improves liver biochemicals such as bilirubin and ALP, it has no effect on progression of disease, health-related quality of life, survival of PSC patients, and finally the requirement for liver transplantation. Also, UDCA does not show any noticeable effect on pruritus, fatigue, or cholangiocarcinoma development (49-52). Nevertheless, the follow-ups and trials of treatment were short as PSC is a slowly progressive disease and trials of 10 years or longer should be included (51). On the other hand, a meta-analysis study by Siddharth Singh et al. indicated that use of UDCA at a low dose (8–15 mg/kg/d) significantly decreased the risk of colorectal neoplasia in patients with PSC-IBD (53).

Studies show that withdrawing UCDA can worsen biochemical test results and pruritus. In a study with 26 patients with PSC who stopped the medication after a period of treatment, a test 3 months after withdrawal of UCDA indicated approximately 61% increase in the average biochemical test and Mayo Risk Score (0.5 point increase from baseline) (54). 

On the other hand, American Association for the Study of Liver Diseases in 2010 issued a guideline recommending against the use of UDCA in the treatment of PSC (55).

Also, in other guidelines, UDCA is not recommended until further data are available on its efficacy and safety. The recommendation upon using UDCA in PSC is to stop UDCA in patients who are already taking it and restart it only if elevation of liver biochemicals such as bilirubin or alkaline phosphatase or a worsening in pruritus was observed and/or the patient experiences symptomatic improvement (54, 56-58).

Higher doses of PSC (28–30 mg/kg/day) results in an improved liver test, but it does not improve survival (59). Furthermore, studies show that high doses (28-30 mg/kg/day) of UDCA in long-term are associated with an increased risk of colorectal neoplasia in PSC patients (60). In a 5-year multicenter, randomized, controlled study by Olsson R et al. it was shown that there was no statistically significant difference in symptoms or quality of life when using a higher dose of UDCA in PSC patients (61). Based on these data, high-dose UDCA should be avoided in patients with PSC. Meanwhile, a derivative of UDCA (24-norursodeoxycholic acid) has shown efficacy in an animal model of PSC (62, 63).

PSC is rare in pregnancy. Studies show that UDCA is safe to be used in pregnancy and shows no fetal effect; nevertheless there are not many reports of interventions in pregnancy and PSC (64, 65).

Combination of UDCA and metronidazole

Metronidazole (MTZ) is an antibiotic that can prevent PSC-like liver damage in vivo models (66). A randomized placebo-controlled study on 80 patients (41 UDCA/placebo and 39 UDCA/MTZ) examined the effect of UDCA and MTZ (UDCA/MTZ) compared with UDCA/placebo on the progression of PSC. A 3-year follow-up showed that patients consuming a combination of UCDA/MTZ had significantly lower serum alkaline phosphatase as compared to those using UCDA with placebo. The New Mayo Risk Score decreased remarkably only in the UDCA/MTZ group. In conclusion, combining MTZ with UDCA in PSC improved New Mayo Risk Score and levels of serum ALP, but no progression was observed in ERCP findings (67). Also, another study found that a combination of UDCA and MTZ tended to improve liver histological stage more than UDCA alone did (68).

Note that in Cockayne Syndrome, administration of MTZ is restricted and can cause fatal acute hepatic failure (69). Also, a study of one case revealed encephalopathy in a patient of Crohn's disease after an IV use of MTZ (70). Additionally, patients taking warfarin should consider warfarin-metronidazole interaction which can result in Intracerebral hemorrhage through the increase in S-warfarin concentrations (71) 

**Vancomycin**

Vancomycin is an antibacterial agent obtained from streptomyces. It is a glycopeptide with a molecular mechanism of bacterial cell wall synthesis inhibition (72). One of the major toxicities of vancomycin is nephrotoxicity, in which demands taking proper medical actions such as therapeutic drug monitoring, changing dosing strategy, and antioxidative therapy for preventing and treating vancomycin-induced acute kidney injury (73).

In small-scale studies, oral vancomycin has shown the possible successful ability in improving liver function tests in patients with pretransplant PSC through immunomodulatory and anti-inflammatory mechanisms (74). Moreover, a recent study on three patients with ulcerative colitis associated to PSC who were treated with 500mg of oral vancomycin twice a day as maintenance therapy, showed a significant clinical improvement and endoscopic remission (a Mayo endoscopic subscore of 0, six months after starting vancomycin). Also, oral vancomycin was tolerated well in these patients (75). In A triple blinded, randomized, placebo-controlled clinical trial on 29 patients with PSC, the efficacy of 125 mg oral vancomycin, four times a day was studied for 12 weeks. This study revealed an acceptable efficacy through a significant decline in the mean level of PSC Mayo risk score and the level of alkaline phosphatase (322.03% and 18.24% respectively) (76).

**Glucocorticoids**

Glucocorticoids are generally the most effective anti-inflammatory drugs available for the treatment of many chronic inflammatory diseases (77). In a study, five months of treatment with USDA was followed by adding glucocorticoid to the regimen in three different groups (two groups with different doses of 3 and 9 mg of budesonide and one group of 10 mg of prednisolone). In the prednisolone group, pruritus, alkaline phosphatase, and IgG decreased significantly, but in the budesonide group, no significant clinical or liver biochemical changes were observed (78). Among the glucocorticoids, budesonide, with a primary role in detoxification and metabolism of bile acids via CYP3A4 and SULT2A1, was well-tolerated in patients with PSC, but it was not found beneficial at doses of 3 mg/day or 9 mg/day (79). 

**Immunomodulators**

Azathioprine (AZT) and its metabolite 6-Mercaptopurine (6-MP), methotrexate (MTX), cyclosporine (CYA), and tacrolimus (Tace) are immune modifier drugs. Both AZT and 6-MP are used extensively in the treatment of IBD, i.e., UC and Crohn’s disease (80, 81). Generally, 6-MP acts as a purine antimetabolite, but AZT which is 6-MPs prodrug, has other modes of action such as prevention of proliferation in cells which is responsible for amplification of immune response by inhibiting several pathways in the biosynthesis of nucleic acids. The pharmacological action of AZT and 6-MP results in alteration of lymphocyte function, reduction in the number of lamina propria plasma cells, and inhibition of natural killer cell function (80, 82). On the other hand, MTX inhibits folate-dependent enzymes. In this regard, studies suggest that the molecular immunosuppression mechanism of MTX is by inhibition of amidophosphoribosyltransferase rather than inhibiting folate-dependent enzymes (83). Further, there are other hypotheses regarding the anti-inflammatory mechanism of MTX such as induction of Ag-specific immune tolerance (84), apoptosis, and clonal deletion of activated peripheral T cells (85). CYA and TACE are calcineurin inhibitors which helps improve the effectiveness of other drugs and their activity. They eventually result in inhibition of T-cell proliferation and generation of antigen-specific CD8+ cytotoxic T-lymphocytes (86). 

The possible inflammatory mechanism of PSC suggests that use of Immunomodulators might be useful in treatment of relapse or for sustaining remission of PSC. A 41-month study of case series showed that progression of PSC in almost all cases stopped and even the condition of the disease improved in some patients using a combination of AZT, prednisolone, and UDCA (87). Despite the potential therapeutic role of MTX in the treatment of PSC, studies have not yet provided a robust results on its effectiveness. In addition, limitations in a clinical study of MTX such as size, duration, and participant heterogeneity have precluded any firm conclusions about the role of MTX in treatment of PSC (88). On the other hand, in a single controlled clinical trial, use of CSA significantly reduced serum alkaline phosphatase, bilirubin, alanine aminotransferase, and gamma globulin in Primary Biliary Cirrhosis (PBC), patients with side effects tolerated well. This suggests that CSA therapy is promising and warrants further evaluation (89). Also, in another study, TACE significantly reduced serum bilirubin and alkaline phosphatase level with no significant side effects in PSC patients and showed the potential role of TACE for the treatment of patients with PSC in feature (90).

**Anti-TNFs**

One of the major classes of immunosuppressants are anti-TNFs, which are the first biological agents in IBD, with studies showing their possible benefit in PSC (91). However, so far, in most studies on anti-TNF drugs, only adalimumab demonstrated a significant decrease in ADA-ALP, while other antagonists of TNF such as pentoxifylline, etanercept, and infliximab have shown very few significant improvements (91). These results finally suggest that anti-TNF biological therapies (excluding adalimumab) are not effective in the treatment of PSC, though further study is required to confirm the effectiveness of both adalimumab and other anti-TNFs (92).

**Vedolizumab**

In addition to anti-TNFs, vedolizumab, which targets alpha beta integrin, has shown controversial results (93-97), and in a most recent study, no significant biochemical respond was observed (98).

**Other drug therapies**

Although UCDA or other treatments reduce the biochemicals of the liver, most long-term studies indicate the fact that in the long run, most of the drug therapies do not reduce risks of mortality and requirement for liver transplantation. Nevertheless, a recent cohort study in Sweden revealed decreased risks of death or need for liver transplantation in patients with IBD-PSC using a combination of statins and azathioprine (99). Elsewhere, in an animal model, curcumin reduced liver damage and cholestasis, inhibited cholangiocyte proliferation as well as TNF-alpha (100). However, clinical trials failed to show any significant improvement in cholestasis or symptoms of the disease (101). Moreover, rifaximin has also been studied as a treatment option for treating PSC. In a study, Tabibian et al. (105) indicated that 12 weeks of treatment with rifaximin did not show any improvement in terms of changes in liver enzymes, fatigue and quality of life of patients with PSC. Although several antibiotics have shown promising results in the management of PSC, other antibiotics such as rifaximin seem to be inefficacious for this indication. 

## Conclusion

PSC is a rare and poorly understood chronic cholestatic fibroinflammatory disease of the liver. It causes severe conditions such as malignancies, cirrhosis, and fibrotic liver diseases. Due to poor understanding of the disease pathways and the small number of participants in clinical studies, proposing a pharmacological treatment for PSC is challenging. Also, factors such as quality of life and the need for liver transplantation need long-term studies. Nevertheless, so far, there have been several promising drugs of pharmacological regimen which help control subclinical - and in some cases clinical - aspects of PSCnt.

## Conflict of interests

The authors declare that they have no conflict of interest.
